# Plant pest and disease lightweight identification model by fusing tensor features and knowledge distillation

**DOI:** 10.3389/fpls.2024.1443815

**Published:** 2024-11-21

**Authors:** Xiaoli Zhang, Kun Liang, Yiying Zhang

**Affiliations:** College of Artificial Intelligence, Tianjin University of Science & Technology, Tianjin, China

**Keywords:** image classification, spatial tensor, knowledge distillation, light weighting, graph convolutional neural networks

## Abstract

Plant pest and disease management is an important factor affecting the yield and quality of crops, and due to the rich variety and the diagnosis process mostly relying on experts’ experience, there are problems of low diagnosis efficiency and accuracy. For this, we proposed a Plant pest and Disease Lightweight identification Model by fusing Tensor features and Knowledge distillation (PDLM-TK). First, a Lightweight Residual Blocks based on Spatial Tensor (LRB-ST) is constructed to enhance the perception and extraction of shallow detail features of plant images by introducing spatial tensor. And the depth separable convolution is used to reduce the number of model parameters to improve the diagnosis efficiency. Secondly, a Branch Network Fusion with Graph Convolutional features (BNF-GC) is proposed to realize image super-pixel segmentation by using spanning tree clustering based on pixel features. And the graph convolution neural network is utilized to extract the correlation features to improve the diagnosis accuracy. Finally, we designed a Model Training Strategy based on knowledge Distillation (MTS-KD) to train the pest and disease diagnosis model by building a knowledge migration architecture, which fully balances the accuracy and diagnosis efficiency of the model. The experimental results show that PDLM-TK performs well in three plant pest and disease datasets such as Plant Village, with the highest classification accuracy and F1 score of 96.19% and 94.94%. Moreover, the model execution efficiency performs better compared to lightweight methods such as MobileViT, which can quickly and accurately diagnose plant diseases.

## 1 Introduction

The variety of crops grown in countries around the world is large and intensive, and according to the world food and agriculture statistical yearbook released by the Food and Agriculture Organization of the United Nations (FAO) in 2023, the global agricultural economy reached $3.7 trillion and employed a population of approximately 873 million people, which accounts for 27% of the global labor force (FAO, 2023). On average, more than 40% of natural losses in agricultural production are caused by plant pests and diseases each year, resulting in a global economic loss of more than 220 billion dollars (FAO, 2022). Thus, once a crop is infested with pests and diseases, it is very easy to cause widespread infection of crops, which seriously affects crop yields and restricts the development of agricultural productivity. However, traditional identification of plant pests and diseases is usually done by experienced plant pathologists or farmers to diagnose the type of leaf pests and diseases. This not only requires a lot of time and effort, but also the process is subjective and limited, which makes it difficult to accurately determine the type of disease and thus leads to the aggravation of plant pests and diseases (Sajitha et al., 2024). Therefore, how to quickly and accurately identify the types of plant pests and diseases is the crucial to ensure the safety and stability of agricultural production, and its research has important theoretical significance and application value.

With the continuous development of smart agriculture and artificial intelligence technology, scholars combined with computer vision related theories to carry out research on plant pest and disease recognition methods. Especially, the research on intelligent plant image recognition based on machine vision and deep learning has achieved better practical results (Rimal et al., 2023). Machine vision methods usually use traditional image processing algorithms or manually designed feature classifiers, which mainly rely on the distinguishing features of different pests and diseases to design the recognition scheme, and are widely used in crop pest detection and classification (Thakuria et al., 2023). However, plant images collected in the natural environment are easily interfered by factors such as light in the environment, which leads to errors in the detection results and inaccurate classification (Zheng and Zhang, 2022). At the same time, with the increasing variety of plant diseases, it is difficult to construct suitable classifiers to distinguish approximate representations by means of manual feature selection. Therefore, traditional machine vision-based pest and disease detection methods are difficult to achieve effective recognition results (Ali et al., 2024). With the continuous development of deep learning in recent years, network models represented by multilayer convolutional neural network (CNN) and attention mechanism have achieved effective results in plant pest and disease recognition (Math and Dharwadkar, 2022). Deep learning based plant pest and disease recognition technique is automated to extract global and contextual features of pest and disease images compared to traditional recognition methods mainly using supervised learning (Roxa et al., 2023). It avoids manual selection of features but extracts richer feature information through autonomous learning, which can cope with diverse crop environments (Sharma et al., 2022). And it can handle massive image data with strong robustness and high accuracy (Shamsul Kamar et al., 2023). In order to further improve the accuracy and training efficiency of the model, techniques such as KD and migration learning are usually combined to fine-tune the parameters of the model to achieve knowledge migration (Khan et al., 2022). Although the above methods can effectively promote the construction of smart agriculture, with the increasing types of plant pests and diseases, as well as the limitations of hardware equipment in the actual application scenarios, there are still some problems that affect the efficiency and accuracy of plant pests and diseases recognition (Manavalan, 2022). 1) The complex structure and large number of parameters of the deep neural network greatly affect the recognition efficiency of the model. 2) The lightweight network model is difficult to mine and fuse the key features, which leads to information loss and thus reduces the accuracy of the model. To address the above problems, we proposed a Plant pest and Disease Lightweight identification Model by fusing Tensor features and Knowledge distillation (MTS-KD) to realize accurate and efficient diagnosis of multiple plant pests and diseases. The specific contributions are as follows.

- Constructed Lightweight Residual Blocks based on Spatial Tensor (LRB-ST). Enhanced the perception and extraction of shallow detail features of plant images by introducing spatial tensor, and reduced the number of model parameters by using depth-separable convolution to improve the diagnostic efficiency.
- Proposed Branch Network Fusion with Graph Convolutional features (BNF-GC). The image super-pixel segmentation was realized by using spanning tree clustering based on pixel features, and the graph convolution neural network was used to extract correlation features to improve the diagnostic accuracy.
- Designed Model Training Strategy based on Knowledge Distillation (MTS-KD). Train the pest and disease diagnosed model by building a knowledge migration architecture, fully balancing the accuracy of the model with the diagnosis efficiency.

The sections of this paper are organized as follows, section 2 focus on introducing and analyzing the current research related to plant pest and disease diagnosis. Section 3 focus on the method proposed in this paper. Section 4 describes the qualitative and quantitative analysis of this paper’s method with other image classification methods to verify the accuracy of the method for plant pest and disease diagnosis. Section 5 discusses the convergence of the method and the configuration of each module to fully justify the method in terms of model construction and parameter selection. Finally, section 6 gives a summary and future research.

## 2 Related work

In order to fully utilize computer or artificial intelligence techniques to assist in pest control, machine learning and deep learning based methods have been proposed for plant image recognition (Cetiner, 2022). While improving the accuracy of more plant pest and disease recognition, it also focuses on lightweight design to improve the diagnostic efficiency. It effectively solves the problem that traditional crop pest and disease image recognition methods rely heavily on manual feature extraction and have poor generalization ability for image recognition in complex backgrounds (Rustia et al., 2022).

The application of machine learning in pest recognition has significantly enhanced the efficiency of pest control. Zhao et al. (2022) and Johari et al. (2022) proposed a multi-step plant adversity recognition method based on hyperspectral imaging and continuous wavelet analysis, used k-mean clustering and support vector machine algorithms to detect abnormal regions of tea tree leaves, and used the random forest algorithm to construct the tea tree discriminant model. Motie et al. (2023) extracted and analyzed spectral vegetation features and used support vector machine (SVM) to classify wheat diseased plants. Bhandari et al. (2023) implemented EfficientNetB5 with a tomato leaf disease (TLD) dataset without any segmentation, and the model achieved high accuracy. Catalkaya et al. (2024) established a KASP (Kompetitive Allele-Specific PCR) analysis method for plant pest identification by designing two forward primers and one reverse primer to enhance the identification accuracy of the algorithm. Pansy and Murali (2023) proposed an unmanned aerial imaging remote sensor for spatio-temporal resolution identification of mango pests and diseases using fuzzy C-mean clustering for diseased leaves and pests, respectively. Despite the deep mining of plant image features, to further enhance the recognition and accuracy, Aldakheel et al. (2024) and Subbaian et al. (2024) applied the YOLOv4 algorithm to plant leaf disease detection. And data enhancement techniques such as histogram equalization and level flipping were used to improve the dataset and effectively enhance the accuracy of plant image disease classification. Zhu et al., 2023 proposed a data evaluation method based on martingale distance and entropy to address the problem of lack of labeling data in intelligent pest identification. This method can filter high value data, thus achieving effective pest recognition performance with small data size. Chodey and Shariff (2023) proposed a Self-Improving Tephritid Swarm Optimization Algorithm (SITSA) to train a pest detection model by selecting the optimal weights and designing a grey scale covariance matrix based feature extraction method to segment the image. Nandhini and Brindha (2024) proposed a new data enhancement technique and feature fusion technique that fuses multi-scale features from global feature extraction network and visual regeneration network to improve accuracy as well as robustness. Although the efficiency of plant pest and disease recognition has been substantially improved, the recognition effect still needs to be improved. This is due to the fact that traditional machine learning methods usually need to select features manually, and their feature representation capability is relatively limited to fully capture the complex structure and information in the image.

Deep learning currently possesses robust feature representation capabilities, enabling it to automatically extract essential semantic information from plant disease and pest images. Li et al. (2024) proposed an interactive bilinear Transformer network, which utilizes fine-grained recognition techniques to realize the types of garden plant diseases. Xia et al. (2023) and Zhang et al. (2023) proposed a pest classification method based on Convolutional Neural Network (CNN) and improved Vision Transformer model, which extracts the features of the objects at different scales and fine-grains, to address the problems of low efficiency of pest classification methods, which are not adapted to large-scale environments. Wei et al. (2022) proposed a multi-scale feature fusion based crop pest classification method (MFFNet), which obtains the deep feature information of pest images through multiple convolutional operations to accurately recognize and classify crop pests. Liu et al. (2024) and Yu et al. (2022) proposed a plant pest type recognition method based on YOLOv5 by introducing modules such as hierarchical classification and attention mechanism, respectively, which effectively avoided the problems of time-consuming, laborious, and inaccurate manual classification. Xiao et al. (2022), on the other hand, combined hyperspectral imaging with deep learning, which designed a spectral feature extraction module through one-dimensional convolution and attention mechanism between spectral channels, effectively utilizing spectral information to improve detection accuracy. On this basis, Cheng et al. (2022) used to recognize the category of tomato diseases in images by fine-tuning the pre-trained model. Qiang et al. (2023) proposed a dual backbone network based pest detection method for citrus leaves in response to the problem that pests on the surface of plants are difficult to distinguish due to their small size and camouflage, which utilizes a single-shot multi-box detector improved by a dual backbone network to enhance the detection accuracy. Dai et al. (2024) and Li et al. (2024) proposed similar deep information feature fusion networks extracting and fusing relevant features from different network layers, respectively, while fusing contextual information at different scales using pyramid-squeezed attention (PSA) to produce better pixel-level attention for improved localization of plant disease areas. Ishengoma et al. (2022) proposed a hybrid convolutional neural network (CNN) model. It was also combined with Unmanned Aerial Vehicle (UAV) technology to build a parallel architecture using two separate models (i.e., VGG 16 and InceptionV3) to realize the identification of plant diseases in large areas. Shafik et al. (2023) proposed an enhanced Convolutional Neural Network (CNN) along the use of Long Short Term Memory (LSTM) using Majority Voting Integrated Classifier for plant disease and pest recognition.

Plant pest and disease identification methods based on artificial intelligence not only enhance the accuracy of disease type identification but also advance agricultural automation and intelligence, helping to mitigate losses caused by pests and diseases. Machine learning-based methods struggle to efficiently extract potential critical features and often require substantial manual labeling efforts. Although deep neural network architectures improve accuracy, they significantly increase model complexity, presenting challenges in parameter training and practical deployment (Li et al., 2023). This complexity results in longer training times and demands greater computational resources, limiting recognition efficiency, particularly in real-time applications. Additionally, the intricacies of hyperparameter tuning and the risk of overfitting pose significant obstacles for practitioners, especially in resource-constrained environments. Although lightweight network models have been proposed to address these issues, fewer parameters often struggle to capture key features in large image datasets. Additionally, noise and other interference present in the images are challenging to filter effectively. These disturbances can obscure important features that are critical for accurate identification of plant pests and diseases. Therefore, how to balance the accuracy and efficiency of plant pest and disease diagnosis methods remains to be solved.

## 3 Methodology

### 3.1 Model framework

In order to improve the efficiency and accuracy of plant pest and disease diagnosis, we constructed a classification model applied to plant pest and disease diagnosis based on a deep learning network architecture, combined with model compression and training methods to achieve accurate identification of various categories of plant pest and disease classes.

**Figure 1:**
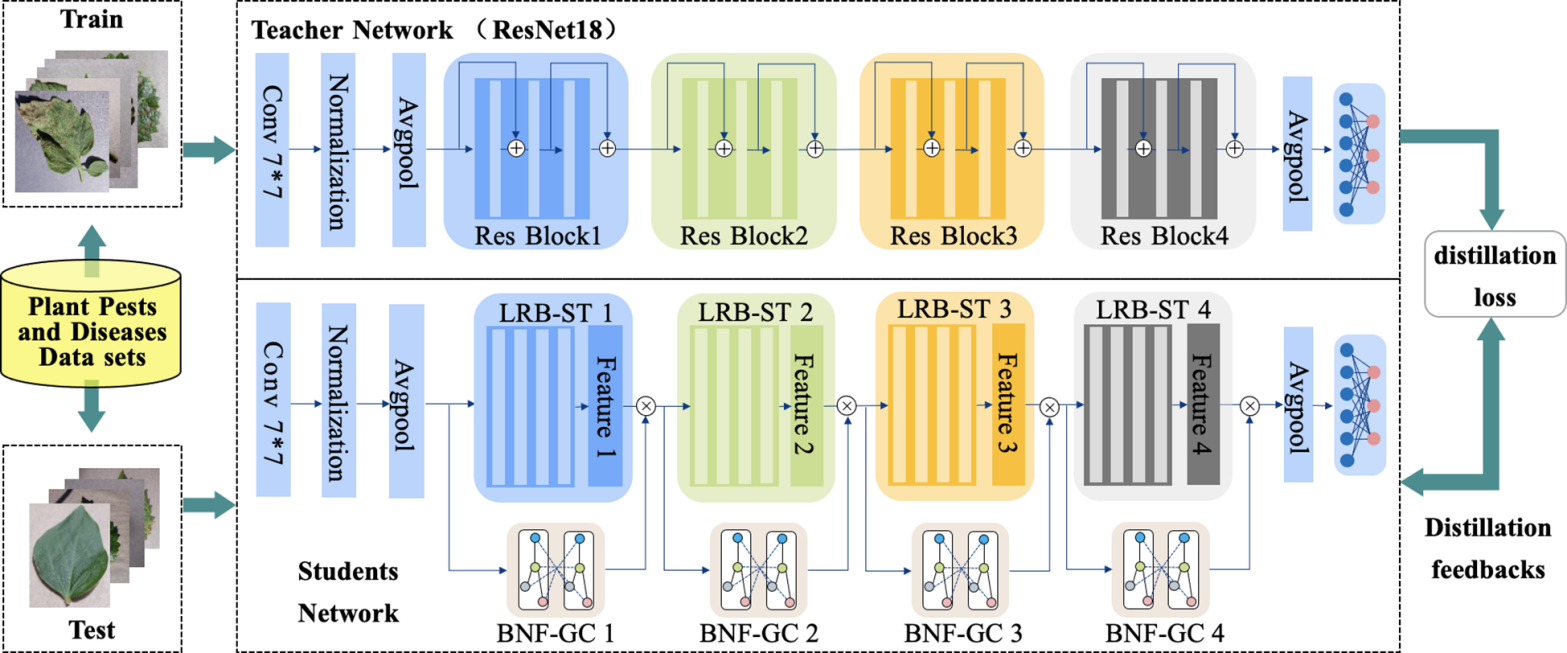
General framework diagram of PDLM-TK.

The PDLM-TK model comprises three primary components, as shown in **Figure 1**. First, a Lightweight Residual Blocks based on Spatial Tensor (LRB-ST) network is designed, which integrates multiple spatial tensors to extract semantic information from plant disease images progressively, from the initial to the final layers. The input plant disease and pest images undergo initial downsampling, followed by four sequential layers of LRB-ST, to capture advanced semantic information about the disease. Next, the Branch Network Fusion with Graph Convolutional features (BNF-GC) is introduced to deeply mine and fuse different levels of residual block features using graph convolution. BNF-GC applies a graph convolutional neural network to the output of each LRB-ST layer, focusing on localized pest and disease information to guide classification. Finally, a Model Training Strategy based on Knowledge Distillation (MTS-KD) is implemented. This strategy utilizes the plant pest and disease dataset to train a teacher network, enabling knowledge transfer to the student network. KD is performed during the student network’s training using the target dataset.

The number of network layers and parameters of PDLM-TK are presented in **Table 1**. PDLM-TK primarily consists of four LRB-STs and BNF-GC as the main feature extraction structure, with a fully connected multi-classification network appended at the end for pest and disease type recognition. Among these, LRB-ST is primarily responsible for extracting key features from pest and disease images, while BNF-GC focuses on mining the correlation features between image regions, thereby achieving a balance between global and local image features. Plant pest and disease images are downsampled through preliminary convolution and pooling layers. Subsequently, feature maps at various levels are deeply mined by LRB-ST and BNF-GC, respectively. It is important to note that LRB-ST utilizes Depth-Separable Convolution (DSC), whereas BNF-GC performs feature extraction using Graph Convolution Network (GCN). The number of output feature map channels for each LRB-ST corresponds to the output feature vector size of BNF-GC. MTS-KD, in turn, is based on PDLM-TK, which serves as the student network for knowledge distillation. The remaining sections of this chapter provide a detailed description of each of these modules.

**Table 1:** Network layers and parameters of PDLM-TK.

Layer Name	Conv_1	LRB-ST _1	LRB-ST _2	LRB-ST _3	LRB-ST _4	Conv_2
		BNF-GC_1	BNF-GC _2	BNF-GC _3	BNF-GC _4	
Output Size	112*112	56*56	28*28	14*14	7*7	1*1
		64	128	256	512	
**Structure**	7*7, 64, stride 2, 3*3, maxpool, stride 2	$\left\{\begin{array}{c} 3*3\text{, }64\\ 3*3,64 \end{array}\right\}$ *2	$\left\{\begin{array}{c} 3*3\text{, }128\\ 3*3,128 \end{array}\right\}$ *2	$\left\{\begin{array}{c} 3*3\text{, }256\\ 3*3,256 \end{array}\right\}$ *2	$\left\{\begin{array}{c} 3*3\text{, }512\\ 3*3,512 \end{array}\right\}$ *2	average, 512, softmax
		(4,25,32)	(4,16,32)	(4,9,32)	(4,4,32)	
**Params[K]**	9.41	31.97	90.15	225.09	1,120	494

The bolded text represents the type and the values represent the best results.

### 3.2 Lightweight residual blocks based on spatial tensor

**Figure 2:**
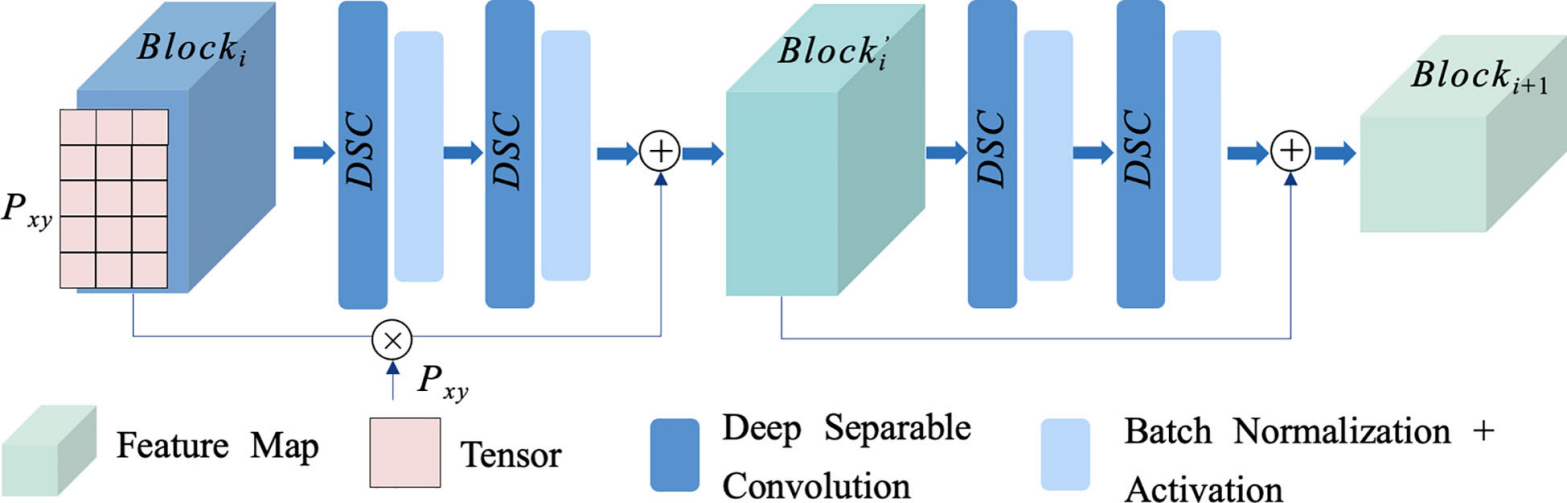
Schematic diagram of LRB-ST.

With the continuous stacking of network layers, the high-level semantic features embedded in images are continuously mined and acquired, but the shallow image semantic features are also easily lost. These features play a crucial role in constructing more complex representations, especially in plant pest and disease recognition, where the texture of some leaves and the color change of damaged areas have a great impact on the final classification results. The residual network enhances the model’s perception of small changes in the input image more effectively by enhancing the underlying semantics. Aiming at the problem that it is difficult to effectively capture and transfer feature information of different dimensions in residual networks, we propose LRB-ST. The principle is shown in **Figure 2**. The semantic information of different dimensions in the input feature graph is mined by defining a trainable spatial tensor. And it is fused with the underlying data as a way to enhance the feature mining ability of the classification model. Meanwhile, in order to improve the classification efficiency of the model, Depthwise Separable Convolution (DSC) is used to replace the traditional convolution operation to reduce the number of model parameters. The specific steps are as follows.

Firstly, the spatial tensor $P_{xy}$ is defined to extract the residual block features. For the input layer *i* feature block $Block_{i}$ , the spatial tensor $P_{xy}$ is defined according to its input size, which is fused by multiplying with the feature maps of each channel of $Block_{i}$ on the original residual connection. The computational procedure is shown in Equation 1. Where DSW represents the depth separable convolution and σ represents the normalization and activation operation on the features.


(1) $$Bloc{k^{\prime }}_{i}=\sigma (DSW(Block_{i}))+P_{xy}*Block_{i}$$

Secondly, depth-separable convolution is used to reduce the number of parameters. Feature extraction is achieved by depth convolution and pointwise convolution, and the input block feature $Block_{i}$ is stacked with the feature fusion result in Equation 1 on an element-by-element basis to obtain the fused feature $Block_{i}^{\prime }$ after two depth-separable convolutions and normalized activation. After that, the feature $Block_{i+1}$ of the residual block is output through the same two depth-separable convolution and used for the calculation of the next residual block. As shown in Equation 2.


(2) $$Block_{i+1}=Block_{i}^{\prime }+\sigma (DSW(Block_{i}^{\prime }))$$

The overall structure of the LRB-ST is depicted in **Figure 2**, comprising two feature superposition processes and one downsampling operation. This study adopts the ResNet18 architecture to facilitate the deep feature extraction of plant pest and disease image data by superimposing four LRB-STs as the backbone network. This approach enables the model to focus on features across different levels while ensuring the effective transmission of deep semantic information. Furthermore, it effectively reduces the number of model parameters, thereby enhancing the classification efficiency of model.

### 3.3 Branch network fusion with graph convolutional features

**Figure 3:**
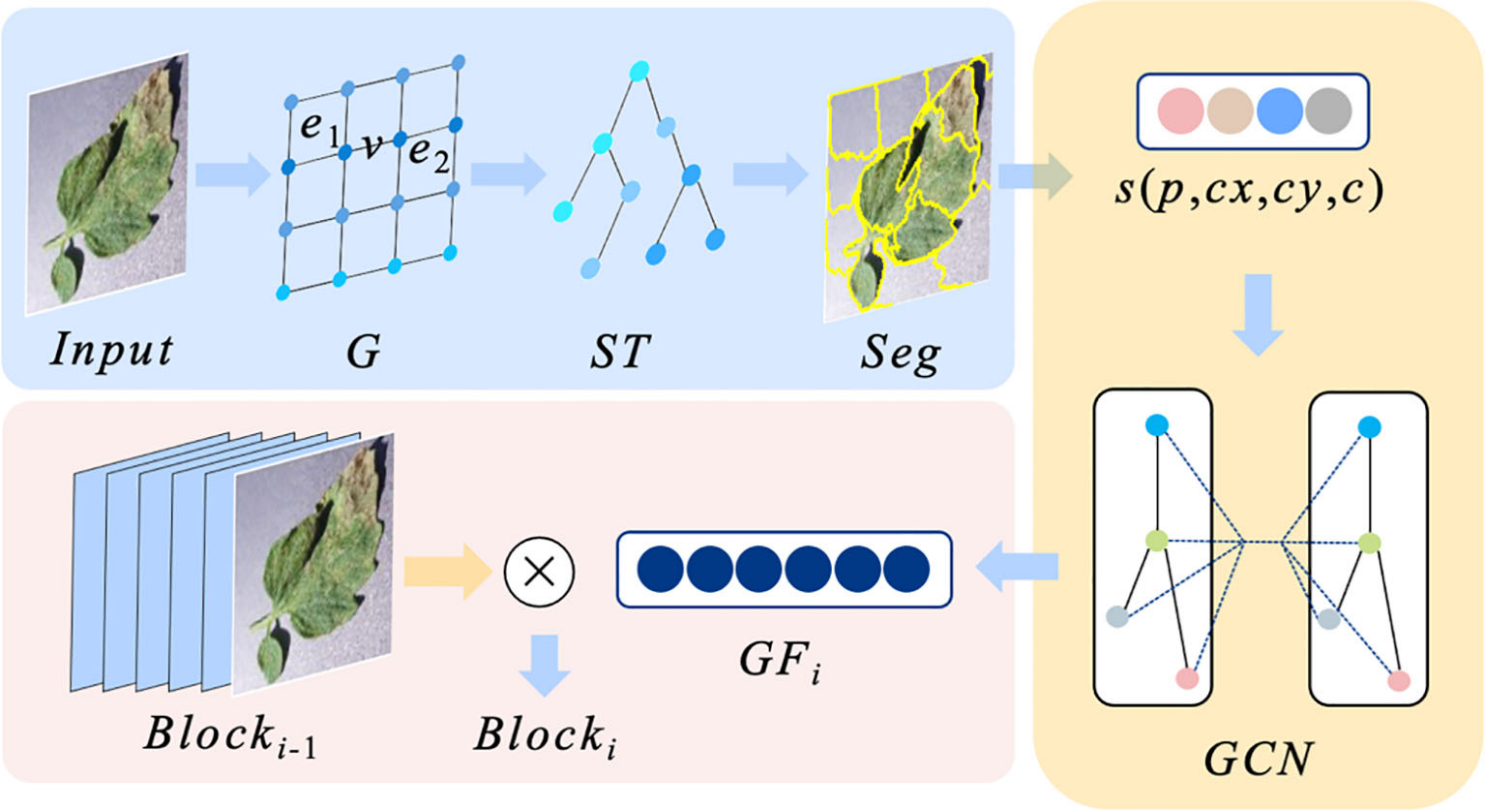
Schematic diagram of BNF-GC.

Since there are factors such as background and noise in the plant image data besides the target region, and the use of deep learning-based classification model can extract key features, but it is still difficult to avoid interference by redundant information, which in turn affects the results of plant pest and disease diagnosis. In order to improve the robustness of the model and mine the correlation features of different regions in the image, we propose BNF-GC, the principle of which is shown in **Figure 3**.

BNF-GC mainly uses super-pixel segmentation to effectively reduce image complexity and extract potential regional features, which can effectively improve the classification performance of the model. Meanwhile, the graph convolutional neural network is used to mine the intrinsic correlation of different regions, which further improves the accuracy of pest and disease diagnosis.

Step1: Construct plant disease image spanning tree ST(v,e,w) . Map the plant image feature data into an undirected graph *G* and represent it as G=(V,E) . Where each pixel point represents a node in the undirected graph and two neighboring pixel points form an edge $e(v_{i},v_{j})$ . Where the weights *w* of the edges are jointly determined by the coordinates $v_{i}(x_{i},y_{i})$ and pixel values $p_{i}$ of the pixel points. The distance between nodes is normalized according to the size of the image M×N and fused with the normalized pixel value features as the weight of each node pixel. The calculation process is shown in Equation 3.


(3) $$w=\sqrt{\left[{\left(x_{i}-x_{j}\right)}^{2}+{\left(y_{i}-y_{j}\right)}^{2}]/(M^{2}+N^{2}\right)}+p_{i}/255$$

Step2: Aggregate the nodes in the region to form a super-pixel segmented image. The nodes are aggregated to form different segmentation regions *R* according to the minimum spanning tree method. The maximum weight $Max(w_{i})$ on the minimum spanning tree in a segmented region indicates the degree of intra-class variation of its internal nodes, while the minimum weight $Min(w_{i})$ between different regions represents its inter-class variation. Therefore, the regions that meet the condition $Max(w_{i})\leq Min(w_{i})$ are merged, after which the inter and intra-class differences are re-compared until the expected number of segmented regions is reached and then stopped, and finally the plant pest image $Seg(R_{1},R_{2},R_{3},\cdots R_{n})$ containing multiple aggregated regions is obtained. The calculation process is shown in Equation 4.


(4) $$Seg(R_{i})=\{\begin{array}{c} \begin{array}{cc} \sum_{i\in R}R_{i}(w_{i}) & Max(w_{i})\leq Min(w_{i}) \end{array}\\ \begin{array}{cc} R_{i}(w_{i}) & other \end{array} \end{array}$$

Step3: Calculate the feature terms of each segmentation region of the superpixel map $Seg(s_{1},s_{2},s_{3},\cdots s_{n})$ . Since the image in each segmentation region contains rich semantic information, this paper selects the pixel mean value $p_{i}$ , the region center coordinate $\left(cx_{i},cy_{i}\right)$ and the number of included pixel points $c_{i}$ in the segmentation region as the superpixel point feature $s_{i}(p_{i},cx_{i},cy_{i},c_{i})$ of each segmentation region. And the feature values of each region are normalized for subsequent feature mining respectively. The calculation process is shown in Equations 5–7.


(5) $$p_{i}=\frac{1}{n}\sum_{j=0}^{n}p_{j}\begin{array}{c} j\in R_{i} \end{array}$$

(6) $$(cx_{i},cy_{i})=\left(\begin{array}{cc} \frac{1}{m}\sum_{i=0}^{m}c_{i}, & \frac{1}{n}\sum_{i=0}^{n}cy_{i} \end{array}\right)\;m,n\in R_{i}$$

(7) $$c_{i}=count(R_{i})$$

Step4: Construct graph convolutional neural network GCN to mine local correlation information. According to the dimension of image features in the segmented region, construct the graph convolution neural network $GCN_{i}$ corresponding to the *i*th residual block, and then mine the regional correlation through multi-layer graph convolution computation to get the feature vector $GF_{i}$ . Finally, the correlation features are multiplied with the output feature map $Block_{i-1}$ of the previous residual block to generate the fusion $Block_{i}$ , which is used for the feature computation of the next residual block. The computation process is shown in Equations 8 and 9.


(8) $$GF_{i}=GCN_{i}(s_{i})$$

(9) $$Block_{i}=Block_{i-1}*GF_{i}$$

The BNF-GC module creates a graph convolutional network branching structure for deep mining of residual block features, which effectively realizes regional relevance feature extraction for plant pest and disease image data. The module enhances the classification performance of the model by enhancing the deep fusion of the underlying semantic information, which emphasizes the main features of the target region and effectively avoids the interference of the background and other information.

### 3.4 Model training strategy based on knowledge distillation

**Figure 4:**
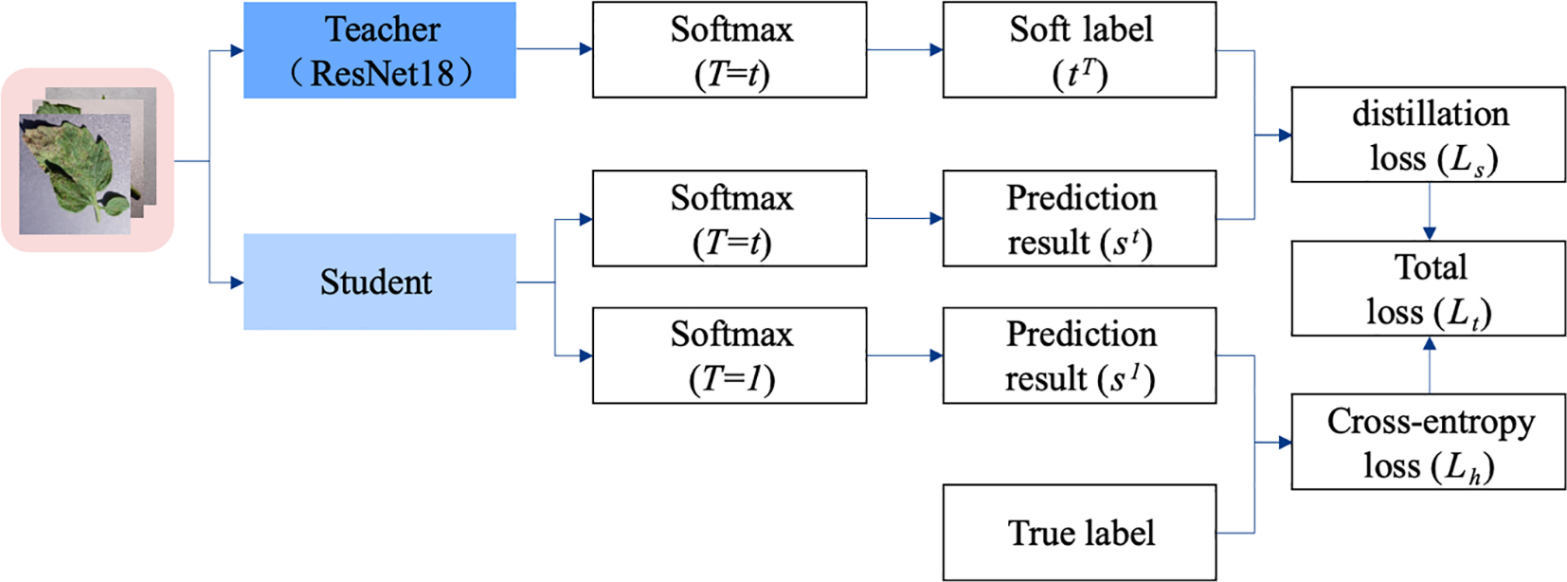
Framework diagram of MTS-KD.

Although existing publicly available datasets contain more images of plant pests and diseases, the lightweight model proposed in this paper contains fewer parameters, which makes it difficult to capture the rich features of the dataset. Especially when the dataset is limited or noisy, it is difficult for the model to learn the implicit knowledge by using only the dataset training. In this regard, this paper proposes MTS-KD, as shown in **Figure 4**. The ResNet18 network is used as the backbone network of the teacher model, and the prediction results and soft labels of the teacher model are used to guide the training of the student model. This approach effectively enhances the model’s learning capacity and improves its generalization ability, which helps mitigate the significant bias present in the plant dataset. As a result, the model’s training efficiency is improved without compromising its high accuracy.

During the training process of the model through MTS-KD, the plant pest and disease images were first categorized according to the training set and test set. After that, they were fed into both teacher-student models for forward computation. The Softmax classification result produced by the teacher model under high temperature *T* is used as soft label $S_{L}$ .

The student model produces the same prediction results after going through the training based on KD. In this case, the Softmax output at the same temperature *T* condition is $L_{s}$ , while the prediction result produced at T=1 is $L_{h}$ . $L_{s}$ in MTS-KD uses Kullback-Leibler Divergence to calculate the relative difference between the predictions of the teacher-student models. In turn, $L_{h}$ uses the cross-entropy loss function to calculate the difference between the results predicted by the student model and the real pest label. Afterwards, the losses of these two components are summed up as the total model loss for optimization and training of the parameters. Where the loss function formulas for $L_{s}$ and $L_{h}$ are shown in Equations 10 and 11.


(10) $$L_{s}=-\sum_{i=0}^{N}s_{i}^{T}\log(t_{i}^{T})$$

(11) $$L_{h}=-\sum_{i=0}^{N}l_{i}\log(t_{i}^{1})$$

Where *N* is the number of total categories in the dataset. $t_{i}^{T}$ refers to the value that the teacher model predicts as *i* after Softmax at temperature *T*. $t_{i}^{1}$ represents the true label prediction result of the teacher model at temperature 1. Similarly, $s_{i}^{T}$ refers to the value of *T* predicted by the student model at temperature *i*, and $l_{i}$ represents the value of the *i*th true label in the total number of categories *N*. The two calculations are shown in Equation 12.


(12) $$p_{i}^{T}=\frac{\exp(P_{i}/T)}{\sum_{N}\exp(P_{N}/T)}$$

$p_{i}^{T}$ in Equation 12 can be used to calculate the predictions for the teacher model and the student model, respectively. Therefore, the total loss $L_{t}$ is defined as shown in Equation 13. Loss weights are employed to balance the influence between soft targets and hard targets from the teacher model during the training process of the student model. In the context of plant pest and disease identification, it is particularly important to emphasize the knowledge derived from the teacher model for training the student network. Where λ is the loss weight for student model training, and (1−λ) is the loss weight for prediction layer distillation.

Model parameter optimization is guided by the Knowledge Distillation (KD) training strategy, which facilitates the transfer of knowledge from the teacher model to the student model. This innovative approach allows the student model to more effectively replicate the feature extraction capabilities of the teacher model, thereby enhancing its ability to grasp complex patterns and nuances in the input images. Furthermore, this knowledge transfer not only improves the classification and recognition performance of the student model but also contributes to training efficiency. When compared to training from scratch, the student model is able to converge to optimal performance levels more quickly and requires less time and resources. Consequently, the implementation of KD not only enhances the capabilities of student models but also simplifies the overall training process, establishing it as a compelling strategy for improving the effectiveness of artificial intelligence in plant pest and disease identification.

## 4 Experimental analysis

### 4.1 Experimental environment and dataset

For the configuration of the experimental environment, the input plant pest and disease image data were randomly clipped into (3, 512, 512) size inputs. The experiments are based on PyTorch 1.11.0 deep learning framework and the operating system of the experimental environment is Ubuntu 20.04. We implement the model code using Python 3.9 programming language and the GPU hardware platform is used with 2 pieces of RTX3080.Adam is chosen as the model optimizer. The batch of input images is 8 at a time. The learning rate is initially set to 0.01, and the number of iterations for model training is set to 120. All models are trained from scratch to ensure the fairness of the comparison results. The configuration of specific parameters is shown in **Table 2**.

**Table 2:** Configuration table of experimental environment and parameters.

Experimental environment	Configuration	Model parameters	Configuration
GPU	RTX3080(10 GB) * 2	optimizer	Adam
OS	Ubuntu 20.04	Batch_size	8
Deep Learning Framework	PyTorch 1.11.0	lr	0.01
programming language	Python 3.9	epoch	120

**Figure 5:**
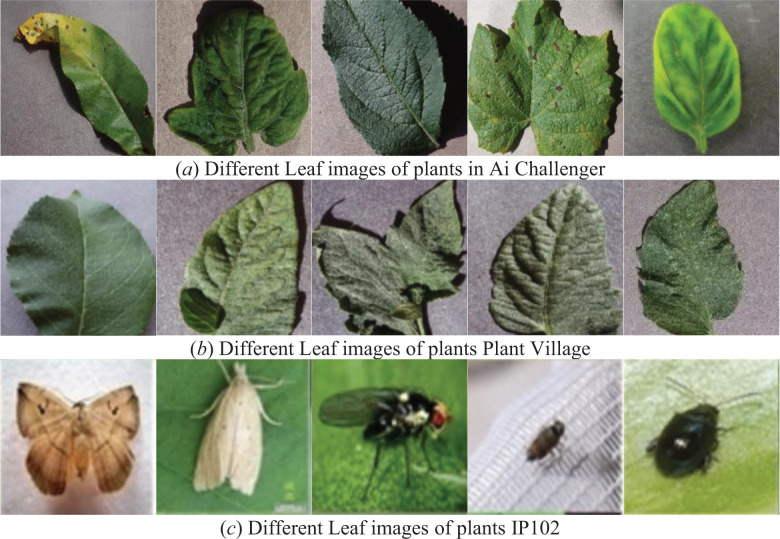
Selected images from the three datasets used for the experiment. As shown in Figures **(A, B)** are five plant leaves from the Ai Challenger and Plant Village datasets, respectively, and the five images in Figure **(C)** are plant insects from the IP102 dataset.

To evaluate the effectiveness of the proposed method, two plant datasets, Ai Challenger (AI Challenge, 2018), Plant Village (Hughes and Salathé, 2015), and an insect dataset, IP102 (Wu et al., 2019), are selected in this paper. The Ai Challenger plant disease identification dataset is shown in **Figure 5A**, which includes 31,718 plant leaf images with 61 categories, including 10 species such as apple and 27 pests. Plant Village dataset is shown in **Figure 5B**, which is labelled by crop pathologists and contains 54309 images with 13 species and 26 crop disease categories. IP102 is a field-constructed large-scale dataset used for pest identification and is shown in **Figure 5C**. It has a total of 75,222 images containing 102 common pests with an average of 737 samples per class. Some images of plant pests and diseases in the three datasets are shown in **Figure 5**. The experimental datasets were divided according to the 8:2 training and testing sets.

### 4.2 Comparative models and evaluation metrics

In order to demonstrate the superiority of the proposed method, the experiment uses five lightweight classification models such as ShuffleNet-V2 (Ma et al., 2018), MobileNet-V3-Large (Howard et al., 2020), EfficientNet B1 (Tan and Le, 2019), MobileViT-S (Mehta and Rastegari, 2022) and Inception v3 (Szegedy et al., 2015) to carry out comparison experiments with PDLM-TK. Since this study belongs to the task of pest and disease image classification, the various models are quantitatively evaluated using Precision (P), Recall (R) and F1 score (F1). And the confusion matrix is drawn according to its evaluation results to visualize the correct classification of the models in each category and realize the visual evaluation of the model performance. Its calculation is shown in Equations 13–15.


(13) $$Precision=\frac{TP}{TP+FP}$$

(14) $$Recall=\frac{TP}{TP+FN}$$

(15) $$F1=\frac{2\times Precision\times Recall}{Precision+Recall}$$

Where TP is the correct image pest category, the number of images that the model correctly predicts as positive instances. FP is the false positive instances of the image, the number of images that the model incorrectly categorizes as positive instances. TN is the number of images that the model correctly predicted as other pest and disease types. FN denotes the number of images that the model incorrectly predicted as other categories when categorizing the correct category.

Considering the large number of pest and disease categories in the plant and insect datasets used, in order to evaluate the diagnostic performance of the proposed methods more comprehensively, the experimental evaluation was statistically analyzed using the Top1 accuracy rate (the accuracy rate of the pest category ranked No. 1 in the model diagnostic results in accordance with the actual results) and the Top5 accuracy rate, respectively.

At the same time, in order to evaluate the diagnostic efficiency of the models, the experiments not only analyzed the performance of the above six models in the three indexes of Acc, P and F1, but also evaluated the number of parameters and the amount of floating-point calculations (GFLOPs) included in the models. In general, the larger the number of parameters included in the model, the lower its training and execution efficiency, and the larger the amount of floating point calculations, the slower the model diagnosis speed.

### 4.3 Quantitative effectiveness evaluation of plant pest and disease diagnostics

The results of the evaluation of Top1 accuracy of six models on three plant pest and disease datasets are shown in **Table 3**. The comparison shows that multiple models have the best classification results on the Plant Village dataset. The reason for this is analyzed to be due to the fact that this dataset contains fewer types of pests and diseases as compared to the other two, and therefore the models classify better on this dataset with less difference in the amount of data. The PDLM-TK method achieved the best P, exceeding the best EfficientNet B1 model by 1.82%, but slightly lower in R and F1 values. This is due to the fact that the composite scaling strategy used by EfficientNet can well utilize the feature extraction ability of the convolutional layer to form a more complete deep learning model network structure. PDLM-TK performs even better on the other two datasets, exceeding the other models by an average of 2.54%, 1.22% and 1.86% on P, R and F1. The experimental results effectively demonstrate the effectiveness of the PDLM-TK method proposed in this paper in plant pest and disease diagnosis. Specifically, the method not only achieves high classification accuracy across various datasets but also showcases superior efficiency compared to traditional approaches.

**Table 3:** Evaluation results of Top1 accuracy for various models.

DataSet	Metrics	Model					
		ShuffleNet-V2	MobileNet-V3-Large	EfficientNet B1	MobileViT-S	Inception v3	PDLM-TK
Ai Challenger	P	86.74	83.21	87.93	82.93	90.42	**92.64**
	R	83.12	81.13	85.10	80.79	87.51	**89.32**
	F1	84.89	82.16	86.49	81.85	88.94	**90.95**
Plant Village	P	90.13	87.20	94.36	92.12	93.62	**95.44**
	R	89.64	85.42	**92.03**	87.48	88.16	90.57
	F1	89.88	86.30	**93.18**	89.74	90.81	92.94
IP102	P	77.75	75.46	78.52	72.13	80.02	**82.87**
	R	72.61	73.60	71.37	70.03	78.46	**79.10**
	F1	75.09	74.52	74.77	71.06	79.23	**80.94**

The bolded text represents the type and the values represent the best results.

In order to evaluate the comprehensive classification performance of the methods and avoid the error that exists in using only Top1 accuracy, as shown in **Table 4**, the evaluation results of Top5 accuracy of various models on the three plant diseases and pests datasets are shown in **Table 3**. According to the experimental results, it can be seen that it is in line with the Top1 accuracy assessment, and the Top5 accuracy assessment results of each model are more accurate compared to the Top1 accuracy. Meanwhile, the PDLM-TK method performed better in classifying pests and diseases on multiple datasets compared to other methods. The highest P, R and F1 values were found on the Ai Challenger and IP102 datasets, and the R and F1 values on the Plant Village dataset were higher than the Inception v3 optimal model by 2% and 0.82, respectively. This shows that our proposed PDLM-TK can realize the diagnosis of plant pests and diseases in a more comprehensive and accurate way.

**Table 4:** Evaluation results of Top5 accuracy for various models.

DataSet	Metrics	Model					
		ShuffleNet-V2	MobileNet-V3-Large	EfficientNet B1	MobileViT-S	Inception v3	PDLM-TK
Ai Challenger	P	89.34	87.11	90.32	85.13	95.71	**96.19**
	R	85.31	84.16	88.49	82.13	91.60	**93.72**
	F1	87.28	85.61	89.40	83.60	93.61	**94.94**
Plant Village	P	94.30	90.16	94.55	93.63	**95.79**	95.20
	R	90.31	88.02	89.90	89.08	90.16	**92.26**
	F1	92.26	89.08	92.17	91.30	92.89	**93.71**
IP102	P	80.13	80.35	82.43	76.64	83.42	**85.51**
	R	76.48	78.31	79.60	74.33	80.02	**80.69**
	F1	78.26	79.32	80.99	75.47	81.68	**83.03**

The bolded text represents the type and the values represent the best results.

### 4.4 Plant pest and disease diagnosis effect visualization and analysis

**Figure 6:**
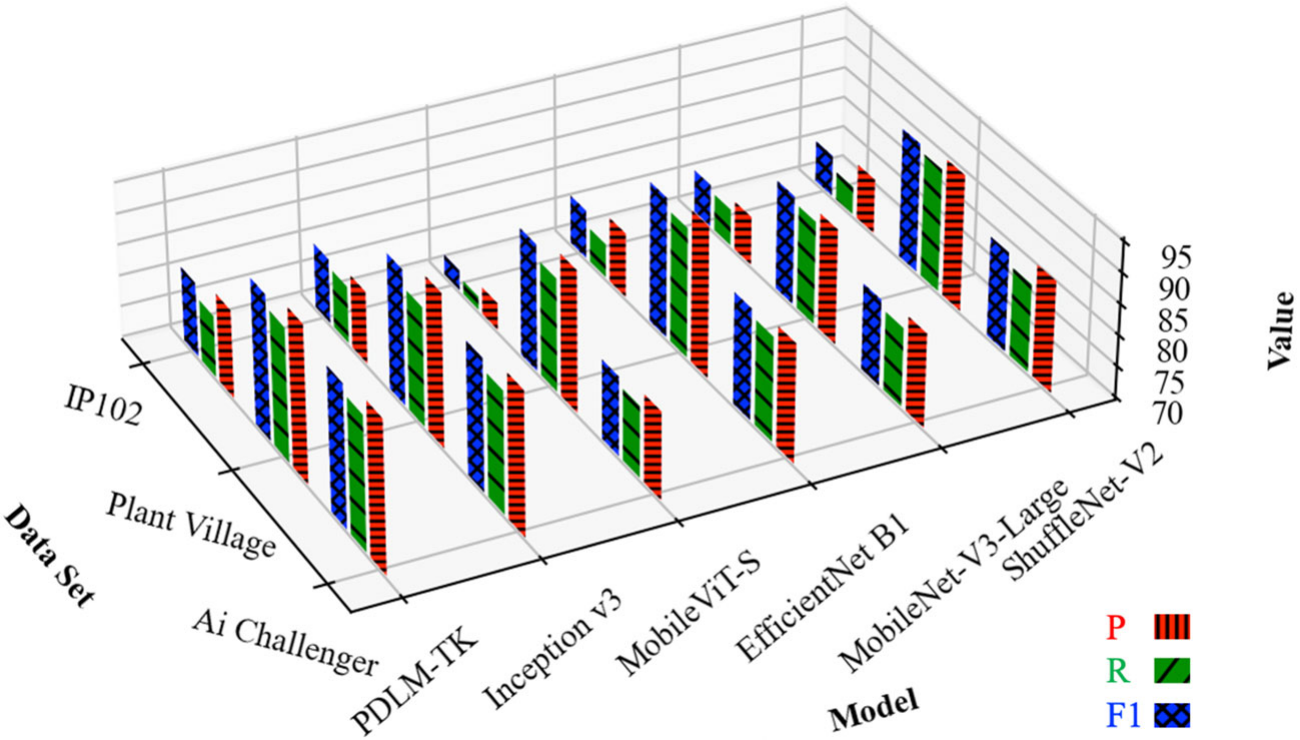
Histogram of Top1 and Top5 average accuracy of various models on three datasets.

As shown in **Figure 6**, the average accuracy histograms of methods such as PDLM-TK on the three plant pest and disease datasets are shown. Analyzing along the direction of the dataset, it can be seen from the height of the histogram that the three colors of the bar represented by PDLM-TK have the highest average on the three datasets. This proves that its combined performance is better on both Top1 and Top5 assessment methods. Meanwhile, according to the column height performance of different models, it can be seen that the heights of the columns of the three colors of PDLM-TK are closer to each other. This indicates that the method has stronger classification accuracy and stability for plant pest and disease images, and can realize more accurate pest and disease diagnosis.

In order to further observe the performance of each model on the test set of plant pests and diseases in a more intuitive way, we utilize the confusion matrix to present the test set classification results of each model. The confusion matrix can be taken in the form of a matrix to summarize the real and predicted categories, and to observe the differences that exist in the prediction results for the classification of different categories. Due to the large number of labels and samples in the plant pest and disease dataset, the numbers in it are no longer displayed, and the performance of each model is only observed and analyzed through the model classification results. Each square in the horizontal and vertical axes of the confusion matrix represents the category corresponding to plant pests and diseases, and the color of each square corresponds to the number of images classified into that region. The darker the color on the diagonal represents the more images that are classified correctly.

**Figure 7:**
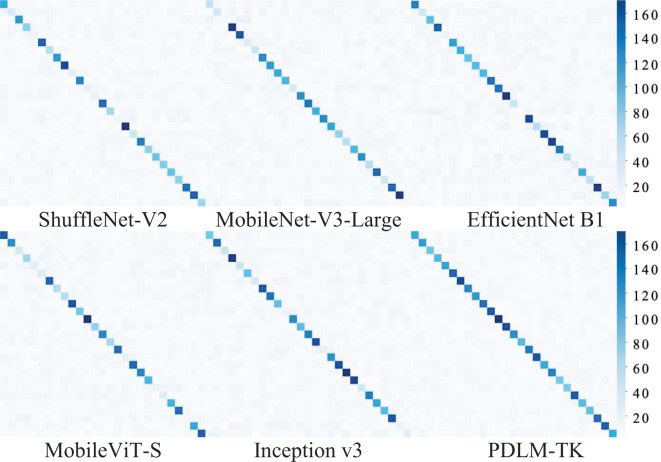
Confusion matrix of different methods on Ai Challenger dataset.

The confusion matrix for each model’s classification prediction for the test set portion of the Ai Challenger dataset is shown in **Figure 7**. From the distribution of squares of different colors in the confusion matrix, it can be seen that the MobileNet-V3-Large and MobileViT-S models have more uniform diagnosis results for different categories of plant pests and diseases, which is mainly manifested by the similar colors of the squares on the diagonal line, but their classification effect is lower than that of the MobileNet-V3-Large and Inception v3 models. Inception v3 has a better classification effect on some kinds of plant pest and disease images, but there is a phenomenon of misclassification of some categories, which is mainly reflected in the lighter color of the squares in the middle of the diagonal of some categories. The confusion matrix corresponding to the PDLM-TK method has a more uniform and saturated color distribution on the diagonal, which shows that its classification effect is more stable.

**Figure 8:**
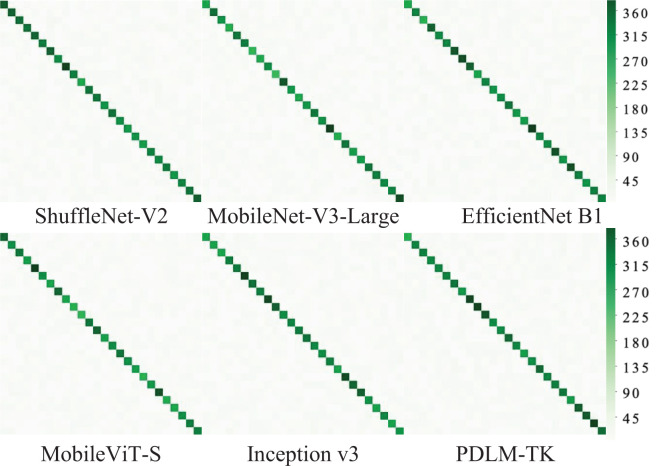
Confusion matrix for different methods on the Plant Village dataset.

The confusion matrix of each model for classifying images on Plant Village test set is shown in **Figure 8**, which is a richer dataset with fewer disease categories. As shown by the color and distribution of diagonal squares, the methods have better classification results. Especially, the two methods, Inception v3 and EfficientNet B1, have the majority of dark-colored squares on the diagonal. The MobileNet-V3-Large method has multiple light-colored squares compared to the two, indicating a partial misclassification. While PDLM-TK has the darkest colored squares compared to the other methods and all of them are distributed on diagonal. It can be fully demonstrated that PDLM-TK has strong classification performance for plant pest disease image diagnosis.

**Figure 9:**
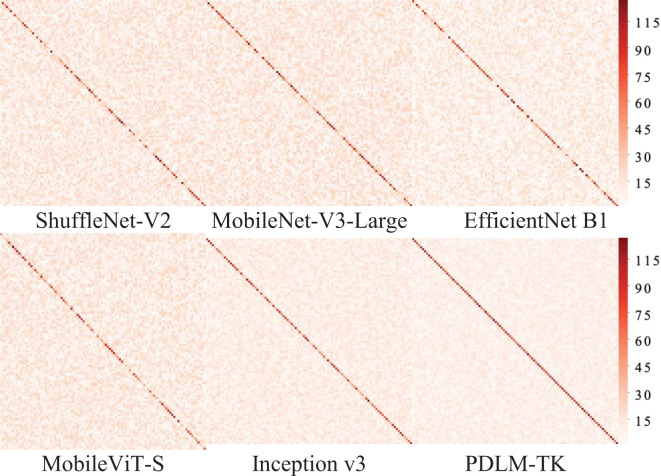
Confusion matrix for different methods on the IP102 dataset.

According to the confusion matrix distribution shown in **Figure 9**, it can be seen that the models are slightly less effective than the other two datasets in classifying insects in the IP102 dataset. The reason is due to the large number of insect species contained in this dataset, and the lightweight classification models are difficult to balance multiple critical features. Among them, two models, EfficientNet B1 and Inception v3, have better classification effects than the other models and can basically realize the discrimination of multiple types of insects. The PDLM-TK method proposed in this paper has the best classification performance compared to the other models because it trains the model by KD, which has a strong feature capturing ability despite the small number of model parameters. In summary, the validation of the plant leaf and insect datasets demonstrates that the PDLM-TK method is able to accurately realize plant pest and disease diagnosis.

## 5 Discussion

### 5.1 Evaluation of model stability and efficiency

Plant pest and disease diagnosis should not only have high accuracy, but also the stability and efficiency of the model is equally important, which is related to the classification effect and diagnosis quality in the practical application of agriculture. Therefore, in this paper, several models are trained on Ai Challenger using the same experimental environment, and their loss functions are plotted as line graphs for analysis. At the same time, the models are ranked according to the number of parameters they contain and their actual execution efficiency, and are compared and analyzed in the form of a table.

**Figure 10:**
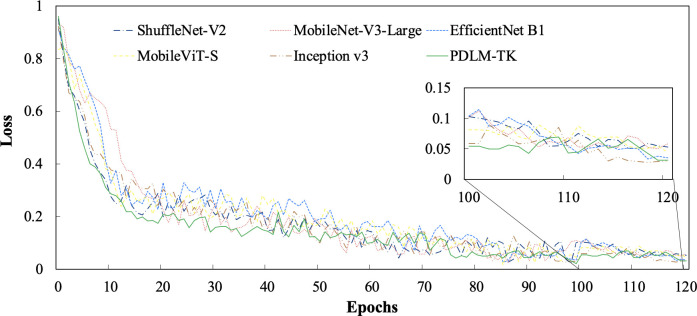
Loss curves for various methods on the IP102 dataset.

As shown in **Figure 10**, the change process of loss when the six methods are trained on the IP102 dataset is shown. From the fluctuation of the folded line, it can be seen that the PDLM- TK method proposed in this paper converges rapidly in the first 20 iteration loops and decreases steadily with the growth of the number of iterations. Notably, after the 110th iteration, the models exhibited stability. While the other methods exhibit similar fold convergence processes, their fluctuations are more pronounced, and their final converged values are higher. This clearly demonstrates that the method proposed in this paper is more stable and achieves faster convergence.

To evaluate the actual running efficiency of each model, this paper uses GFLOPs and Params metrics to measure the execution speed of the models. As shown in **Table 5**, EfficientNet B1 has the smallest amount of computation, and PDLM-TK is only 0.1 behind it in the second place. As shown by the parameter count, PDLM-TK contains the least number of parameters, followed by MobileNet-V3-Large. Combining the evaluation indexes such as the accuracy of each method, the PDLM-TK method achieves higher accuracy through less time, and is able to better balance the efficiency and accuracy of the model, compared to other models both in diagnostic accuracy and efficiency.

**Table 5:** Statistical results of parameters and computational efficiency from various models.

Metrics	ShuffleNet-V2	MobileNet-V3-Large	EfficientNet B1	MobileViT-S	Inception v3	PDLM-TK
GFLOPs	0.56	0.23	**0.6**	1.75	9.62	0.7
Params[M]	5.6	4.3	6.6	5.1	24.7	**1.97**

The bolded text represents the type and the values represent the best results.

### 5.2 Assessment of KD effectiveness in teacher networks

As presented in **Table 6**, the evaluation results of the Top1 accuracy for PDLM-TK when different teacher networks were utilized across three plant pest and disease datasets are shown. The comparison indicates that using ResNet18 as the teacher network yielded superior overall results, surpassing the ImageNet model by 0.63, 1.14, and 0.9 in the P, R, and F1 metrics, respectively. This improvement is attributed to the structural similarities between PDLM-TK’s backbone, which is derived from the residual blocks of ResNet. It can thus be concluded that knowledge migration is more effective when the chosen teacher network has a similar model structure, enhancing the effectiveness of the KD process.

**Table 6:** Top1 accuracy evaluation results of different teacher networks.

Teacher Networks	P		R		F1	
	ResNet18	ImageNet	ResNet18	ImageNet	ResNet18	ImageNet
**Ai Challenger**	92.64	91.27	89.32	88.36	90.95	89.79
**Plant Village**	95.44	96.62	90.57	90.77	92.94	93.60
**IP102**	82.87	81.13	79.10	76.43	80.94	78.71
**Average**	**90.31**	89.67	**86.33**	85.18	**88.27**	87.37

The bolded text represents the type and the values represent the best results.

### 5.3 Network grad-CAM visualization

**Figure 11:**
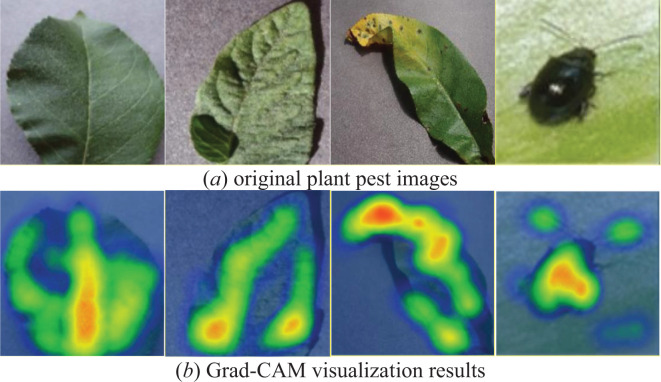
Plant disease and pest images used Grad-CAM visualization results, figure **(A)** shows the plant leaves in the dataset and figure **(B)** shows the results visualized using Grad-CAM.

To better assess the PDLM-TK model’s ability to learn the characteristics of plant pests and diseases, we predicted a portion of the test set data for each disease and visualized the results using Grad-CAM. The visualization outcomes are presented in **Figure 11**. In this study, the last layer of the PDLM-TK model was selected as the feature visualization layer, with the heat map superimposed on the original image. The **Figure 11A** displays the original plant pest images, while the **Figure 11B** shows the weighted visualization results.

Upon examining the visualization results, we observed that the PDLM-TK model not only accurately predicted the classification of each disease but also successfully identified the key regions corresponding to various plant disease locations or pests. Additionally, it was noted that the model paid less attention to irrelevant and complex backgrounds surrounding the diseased leaves, instead concentrating on the characterization of disease features and pests during the feature selection process.

### 5.4 Ablation experiment

Based on the existing plant disease and pest classification model structure we designed the PDLM-TK model, which contains several modules to realize feature extraction, mining and model training respectively. Among them, residual blocks integrating multiple LRB-STs are designed to enhance the deep extraction of semantic features of different dimensions and to improve the model classification accuracy, which extracts the semantic information of different channels in plant disease images layer by layer. For the redundant information interference in the image, we proposed BNF-GC, which deeply mines the residual block features by graph convolution in order to focus on the disease features. We utilize the plant pest and disease dataset to train the teacher network and construct MTS-KD to realize knowledge transfer to the student network. To fully evaluate the main role played by each module, we designed ablation experiments based on the architecture of unused modules (Basic). Finally, they are evaluated and analyzed on Ai Challenger using P, R and F1 respectively.

As shown in **Table 7** for the results of the ablation experiments of each module. “✓” stands for the blocks being selected, in the first four sets of experiments the Basic model (ResNet18) without several modules has the lowest evaluation scores, while the effect of using MTS-KD is higher than the other two types of modules. This proves that each module plays a role in improving the model classification effect, especially the effect of MTS-KD improves the model classification performance more. From the experimental results of groups 5 to 7, it can be seen that BNF-GC and MTS-KD have the best R when they are paired together, which is higher than the effect of group 8 when multiple modules are used at the same time, but it is slightly lower in terms of P and F1 values. The reason for this is that the BNF-GC module is able to better balance the feature information between different levels, but due to the similarity of some plant pests and diseases in terms of the types of performance, it is difficult to distinguish the real categories by only emphasizing the attention to the same representations, which leads to the easy occurrence of the misclassification phenomenon. With the addition of LRB-ST enables the multilayer channel semantic information to be better mined, which is used to enhance the model’s capture of plant pest and disease characteristics for each category. Therefore, a combination of multiple evaluation indexes leads to the best classification performance of the PDLM-TK method incorporating the three modules.

**Table 7:** Evaluation results of ablation experiments.

No.	Blocks				Metrics		
	Basic	LRB-ST	BNF-GC	MTS-KD	P	R	F1
1	✓				76.43	73.21	74.79
2	✓	✓			82.64	80.3	81.45
3	✓		✓		83.22	79.67	81.41
4	✓			✓	85.34	81.27	83.26
5	✓	✓	✓		86.42	83.13	84.74
6	✓		✓	✓	92.35	**89.47**	90.89
7	✓	✓		✓	91.42	88.74	90.06
8	✓	✓	✓	✓	**92.64**	89.32	**90.95**

The bolded text represents the type and the values represent the best results.

## 6 Conclusion

To address the issues of low efficiency and accuracy in plant pest and disease diagnosis, the capabilities of tensor and graph deep learning in feature mining are fully utilized. The model network structure is optimized by integrating knowledge distillation (KD) and other techniques, resulting in the proposal of a Plant Pest and Disease Lightweight Identification Model (PDLM-TK) that fuses tensor features and KD. First, a lightweight residual block based on spatial tensor is constructed to enhance the perception and extraction of shallow detail features of plant images by introducing spatial tensor, and depth separable convolution is used to reduce the number of model parameters to improve the diagnostic efficiency. Secondly, a branching network incorporating graph convolutional features is proposed to realize image super-pixel segmentation by using spanning tree clustering based on pixel features, and graph convolutional neural network is used to extract correlation features to improve diagnostic accuracy. Finally, a model training strategy based on KD is designed to train the pest and disease diagnosis model by building a knowledge migration architecture, which fully balances the accuracy and diagnosis efficiency of the model.

In order to verify the diagnostic performance of the model in plant diseases and pests, we carried out experiments on three plant disease and pest datasets, including Plant Village, and the results proved that PDLM-TK had the best performance in terms of classification accuracy and efficiency, and was able to realize fast and accurate diagnosis of plant diseases. Although the method achieves better plant disease and pest classification results, the performance of the model in the face of some small-sample disease and pest datasets remains to be examined, and in the future, we can further increase the number of disease and pest species as well as datasets with different sample set sizes for testing, so as to further enhance the model’s value for practical application. In the subsequent study, we will further integrate real farming environments within the experimental field, collect data on a variety of plant diseases and pests, and assess the model’s performance in varied deployment settings and on smaller-scale farmland.

## Data Availability

The datasets presented in this study can be found in online repositories. The names of the repository/repositories and accession number(s) can be found in the article/supplementary material.
